# Influence of Carbon Fibre Addition, Ultrasonication and Vacuum Processing on the Mechanical and Conductive Properties of Expanded Graphite Polyester Resin Composites

**DOI:** 10.3390/polym18060731

**Published:** 2026-03-17

**Authors:** Divan Coetzee, Juan Pablo Perez Aguilera, Akshat Tegginamath, Jakub Wiener

**Affiliations:** 1 Department of Material Engineering, Faculty of Textile Engineering, Technical University of Liberec, 46117 Liberec, Czech Republic; 2 Institute of New Technologies and Applied Informatics, Faculty of Mechatronics, Informatics and Interdisciplinary Studies, 46117 Liberec, Czech Republic; 3 Department of Machine Parts and Mechanism, Faculty of Mechanical Engineering, Technical University of Liberec, 46117 Liberec, Czech Republic

**Keywords:** expanded graphite, carbon fibre, conductive composite, polyester resin, vacuum composite processing, ultrasonication

## Abstract

Polyester resin composites containing expanded graphite often exhibit reduced mechanical strength due to the porous structure of the filler. The aim of this study was to enhance mechanical performance without compromising electrical behaviour. Although carbon fibre and expanded graphite are chemically identical carbon allotropes, their distinct morphologies motivated the use of carbon fibre to reinforce expanded graphite-filled polyester composites. To examine the role of expanded graphite porosity, ultrasonicated EG was used to produce exfoliated, lower-porosity particles, while vacuum processing was applied to remove entrapped air prior to curing. Adding 0.5–5 wt% milled carbon fibre increased electrical conductivity by up to three orders of magnitude relative to neat polyester while maintaining 70–80% of the original specific strength at moderate fibre contents. Ultrasonicated EG reduced tensile strength by more than 50% at 5 wt% loading and decreased conductivity due to additional grain boundary formation. Vacuum-processed EG not only provided slight mechanical enhancements but also significantly improved electrical properties by lowering surface resistance by 6–10 orders of magnitude, reaching the tens-of-Ω range at 3–5 wt% EG. This performance is comparable to previously reported conductive EG/polymer systems, which exhibit surface resistances of 10–10^2^ Ω at 5 wt% EG. This systematic comparison offers practical guidelines for balancing conductive percolation and mechanical reinforcement in expanded graphite polyester composites.

## 1. Introduction

Polyester composites are used for various applications due to their desired properties, such as strength and reinforcement capability, but the polymer is thermally and electrically insulating. Expanded graphite (EG) can enhance the thermal and electrical conductivity of resin polymers. This is typically performed by blending thermally expanded graphite into a resin matrix [1,2,3,4]. Expanded graphite is an excellent alternative compared to using graphene or graphite since it has better particle dispersion as graphene tends to agglomerate. When properly exfoliated, expanded graphite can have better mechanical properties due to the larger surface area forming better matrix bonds. This larger particle surface area also increases the thermal conductivity, which is enhanced by the continuity of the graphite network. Expanded graphite, although more expensive than its precursor, graphite, is still considerably cheaper than graphene. The aforementioned advantages also relate to expanded graphite’s ease of processing [5,6,7].

**Figure 1:**
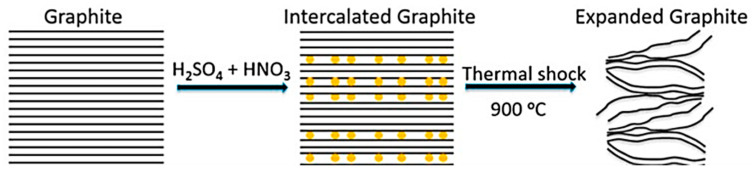
Thermal expansion process to produce expanded graphite [10] (image used under Creative Commons 4.0 licence).

**Figure 2:**
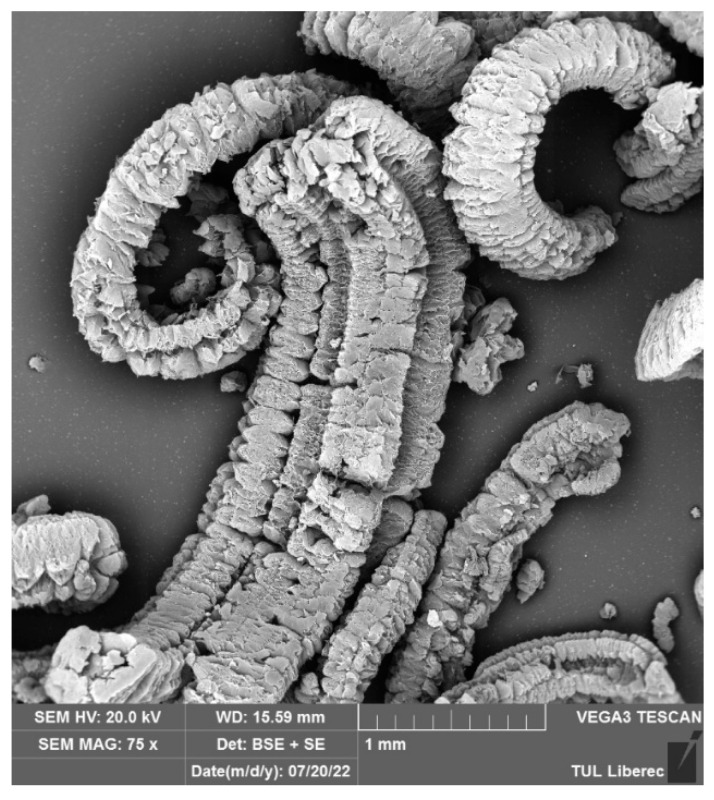
SEM image of expanded graphite [11] (image used under Creative Commons 4.0 licence).

Thermal expansion is the most common method used to produce expanded graphite. Natural graphite flakes are used as the starting material, followed by intercalation using hydrogen peroxide and acids such as sulphuric or nitric acid to form expandable graphite. This is then washed to remove excess intercalant. The dried expandable graphite is then rapidly heated to temperatures around 900 °C. This causes the intercalant to rapidly convert to gas, expanding the graphitic layers to 300 times the original interlayer distance. This expansion in the direction perpendicular to the graphene sheet planes forms a worm-like particle structure with large porosity. The thermal expansion process is illustrated in Figure 1, and a scanning electron microscope image of the particles is presented in Figure 2. The expanded graphite can also be processed into other non-particle forms, such as flexible graphite foils and tapes [8,9].

Previous experiments have shown that expanded graphite tends to decrease the mechanical strength of viscous resin samples. This was attributed to the filler material’s porous nature, which trapped air in the composite structure. Due to the high viscosity of the polyester resin, this was more prominent. In the study, other samples with lower matrix viscosity and heavier processing did not exhibit the problem to the same degree, and the expanded graphite acted as a reinforcement [12]. These observations were in line with other experiments performed on exfoliated expanded graphite [13,14,15]. Three different approaches were made to recover mechanical losses. The first was introducing milled carbon fibre into the matrix since it is a known reinforcement. The second was ultrasonicating the expanded graphite to exfoliate and remove the air trapped inside the particles. Thirdly, vacuum processing was used, which produced the samples by placing them in vacuum before curing them to remove trapped air in the matrix.

Ultrasonication was selected as the preferred method for exfoliating expanded graphite because it is more scalable, given the material’s large porosity, which can limit alternative techniques like ball milling. This process involves using high-frequency sound waves to exfoliate the expanded graphite in a suspension through cavitation. Initially, small bubbles form between the cavities in the graphite layers due to ultrasonic waves passing through the liquid, causing pressure fluctuations. These bubbles then rapidly collapse, generating intense local temperatures and pressures that produce shockwaves, which then initiate layer separation. During this process, the microjets generated by the shockwaves have sufficient energy to overcome the Van der Waals forces holding the graphite layers together, resulting in exfoliation. This is followed by stabilisation, where the particles either agglomerate or are kept separated by stabilising solvents or agents such as surfactants [16,17,18,19,20].

Most prior studies on expanded graphite polymer composites have focused either on optimising electrical or thermal percolation in a single matrix system or on improving one property class at a time. For example, Ramanujam et al. reported processing-route-dependent percolation thresholds of 0.5–3 wt% expanded graphite in polyphenylene sulphide, but did not quantify the associated trade-offs in mechanical properties. Similarly, expanded graphite epoxy and pitch composites have been engineered for low electrical thresholds down to 1.5 wt% EG and high EMI shielding, again with only limited mechanical characterisation [21,22,23,24]. In contrast, the present work systematically compares three distinct routes—milled CF addition, ultrasonication of EG and vacuum processing—within the same viscous polyester matrix, and analyses their coupled effects on tensile strength, stiffness, density-normalised properties and electrical percolation using a unified ANOVA and regression framework. This enables the direct mapping of the strength–conductivity design space as a function of both filler type and processing, which is believed to address a critical knowledge gap in EG-based thermoset composites.

## 2. Materials and Methods

### 2.1. Materials

The expanded graphite used was produced by Sorbetin s.r.o., Vsetín, Czech Republic, using the thermal shock method with sulphuric acid GIC. Polyester resin UN1866 (Havel Composites CZ s.r.o., Svésedlice, Czech Republic, viscosity 400–600 mPa·s at 20 °C) was used, along with the manufacturer-recommended hardener, for the experiment. Milled carbon fibre (CF) was supplied by Easy Composites (Stoke-on-Trent, UK) with a nominal length of 100 ± 50 µm and a diameter of 7–10 µm, giving a moderate aspect ratio suitable for dispersion in viscous thermosets. The unprocessed EG was placed in a 300 mL beaker, half-filled with EG, and topped up with distilled water to produce the ultrasonicated expanded graphite. It was then ultrasonicated for 1 h at 45 Watts at 40 KHz until no more porous EG was floating on the surface. Temperature control was set at 78 °C, after which the samples were removed from the ultrasonicator and cooled in an ice bath before continuing for the specified time. This was then filtered and dried in an oven at 80 °C for 30 min.

### 2.2. Sample Preparation

For all formulations, 60 g of resin was premixed with the appropriate amount of CF and/or EG using a mechanical overhead stirrer at 500 rpm for 5 min, followed by the addition of 2 wt% MEKP hardener and a further 2 min of mixing. The mixtures were then allowed to rest for 10 min at room temperature to permit initial bubble rise, before casting into moulds in the shape stipulated in the standard ISO 527-2 using Type 1A specimens (gauge length 50 mm, width 10 mm, thickness 4 mm) and illustrated in the Supplementary Materials and curing at 60 °C for 6 h [25]. This work utilised 1%, 3%, and 5% expanded graphite (EG), combined with 0.5%, 1%, 3%, and 5% milled carbon fibre (CF) to produce the hybrid samples. Samples were labelled according to filler type and loading, where xCFxEG denotes x wt% carbon fibre and x wt% expanded graphite. Samples containing ultrasonicated EG were labelled as xuEG, where x represented the specific ultrasonicated EG percentages of 0.1%, 1%, 3%, and 5%. For vacEG samples, the resin–filler mixture was cast into the moulds and then placed in a vacuum chamber at 400 mbar absolute pressure (i.e., 0.4 bar) for 24 h at room temperature prior to thermal curing. Vacuum was therefore applied in the moulds, after mixing but before polymerisation, with the intention of removing both bulk entrapped air and air trapped within the EG pore structure. These samples were designated as xvacEG, where x indicated 1%, 3%, and 5% expanded graphite, respectively.

### 2.3. Measurement

For each composition, *n* = 5 tensile and resistivity specimens were tested. For resistivity, 10 readings were taken on each specimen and averaged to obtain a specimen-level value; the specimen means were then averaged to give the composition means plotted. All samples were visually examined using a scanning electron microscope (SEM) (Vega 3 Tescan, Brno, Czech Republic). Electrical resistivity measurements were performed according to ASTM D257 using the two-probe method with a 1.0 cm spacing [26]. The samples xuEG and xCF were measured on Hewlett-Packard 4339B, Japan, while the samples xCFxEG and xvacEG were measured on an Agilent 53131A 225 MHz, Republic of Korea, universal counter due to their higher electrical conductivity. The 2W method was chosen because of the samples’ thin structure, allowing easier measurement. Measurements were conducted under conditions specified in the data sheets. Uniaxial tensile tests were performed on Tiratest 2300, TIRA, Schalkau, Germany according to ISO 527-2 at a crosshead speed of 2 mm/min, and engineering stress–strain curves were recorded [25]. The results were validated through numerical modelling using ANSYS 2024. XRD analysis was carried out using a Philips Panalytical PRO MPD, Almelo, The Netherlands, with a copper source and a PIXcel 1D detector (Panalytical, Almelo, The Netherlands). The initial and final 2-theta angles were 5° and 50°, respectively. Each sample was measured twice, and the average of the two datasets was used to generate the diffraction spectra.

### 2.4. Statistical Processing

Raw tensile data were processed for outliers using a Q-test at α = 0.05. One-way ANOVA was performed on the group means of the five main material families (neat polyester, CF-only, uEG, vacEG and CF/EG hybrids) for each mechanical response (max load, tensile modulus, specific strength, specific modulus) and for log_10_ (surface resistivity). The ANOVA was implemented via both the classical F-test and linear models with Type II sums of squares, treating each composition as a single observation at the formulation level. Where the ANOVA F-test indicated a significant group effect at α = 0.05, Tukey’s HSD post hoc test was applied to identify significantly different pairs. Full ANOVA tables, including F-values, degrees of freedom and *p*-values, as well as pairwise Tukey HSD results, are reported in the supplementary analysis. All statistical analyses were performed using SciPy v1.13.1 and Statsmodels v0.14.4.

## 3. Results and Discussion

### 3.1. Scanning Electron Microscopy

**Figure 3:**
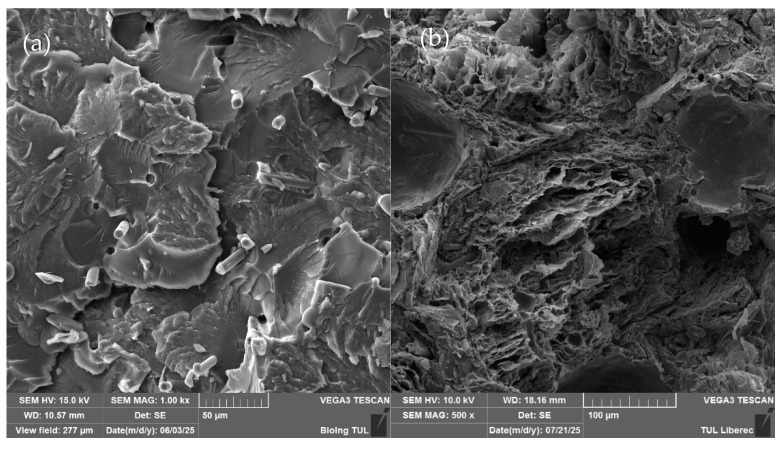

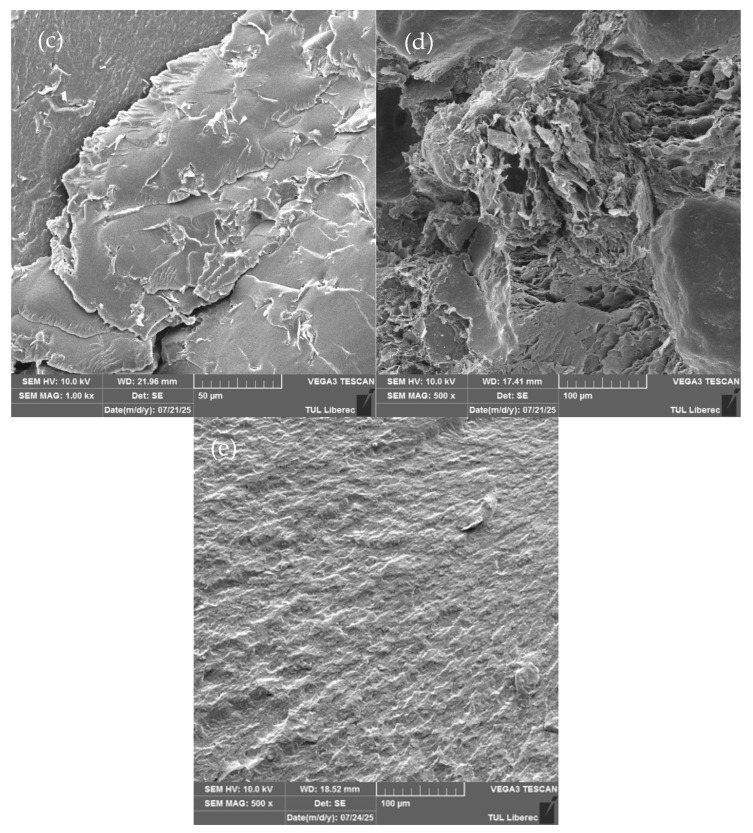
SEM images of samples: (**a**) 5%CF, (**b**) 5% CF 5% EG, (**c**) 5% uEG, (**d**) 5% vacEG, and (**e**) polyester resin with no filler.

SEM was utilised to investigate filler dispersion, interfacial bonding, and the microstructural origins of mechanical behaviour, as illustrated in Figure 3, which displays a cross-section of the fracture point of the tested samples. Visually, the particles are well dispersed throughout the samples after curing. For a clearer illustration, the samples with the highest particle content are displayed. In Figure 3a, carbon fibre rod-like structures are visible, along with some holes where the CF filler was pulled out during fracture. This indicates weak interfacial bonding between the CF filler and the matrix. From this image, it was clear that, after mixing, the fibres adopted a random orientation in the matrix, which is expected to negatively affect the material’s axial mechanical properties, even though dispersion was uniform. Figure 3b presents the combined carbon fibre and expanded graphite, with a similar fracture pattern to the CF sample, where the filler provided some reinforcement before the interfacial bonds failed. The porous EG contributed very little reinforcement; particle fracturing was observed, attributed to their porosity. Figure 3c shows the ultrasonicated expanded graphite sample, with fine graphene-like sheets visible throughout the structure. The particles are well dispersed, with the polymer matrix surrounding most of them. This likely reduces electrical conductivity, as polyester resin is an insulator. The vacuum-processed expanded graphite sample exhibits a more compact structure than in Figure 3b, with an enhanced particle–polymer interaction reducing free space within the structure. Nonetheless, some trapped air remains, though significantly less at higher loadings compared to non-vacuum-processed samples. The EG particles are still the weakest point in the composite, as shown by their breakage. Figure 3e illustrates the breaking point of the unfilled PES resin sample showing a clear break as there were no other elements effecting the fracture point. It is clear that directly adding graphite traps air within the structure, which is expected to negatively impact both mechanical and electrical properties due to increased insulating effects.

Beyond gross dispersion and porosity, the SEM images also provide insights into interfacial adhesion mechanisms that help explain the tensile results. In the CF-only sample (Figure 3a), numerous fibre impressions and cavities are visible at the fracture surface, indicating that fibres were pulled out rather than cleanly broken. This suggests an interfacial shear strength that is sufficient to transfer load up to a point, but not high enough to force fibre fracture, consistent with the moderate specific strength and stiffness observed for CF-only composites. Similar pull-out and debonding are seen in the CF/EG hybrids (Figure 3b), where the presence of EG exacerbates stress concentration at fibre ends and leads to mixed-mode failure. In contrast, the uEG sample (Figure 3c) exhibits fine graphene-like sheets more conformally coated by the resin, with fewer large voids; this morphology supports partial strength retention but, because the conductive pathways are more tortuous and resin-filled, is detrimental to electrical conductivity. The vacEG microstructure (Figure 3d) shows a denser, more compact EG network with fewer large pores, but still with evidence of internal voids and brittle particle fracture. Such a structure explains the high-specific modulus, stiff, low-density EG “skeleton”, combined with a pronounced loss in tensile strength due to crack initiation within and around the brittle, porous graphite domains. These observations agree with broader findings in EG- and graphene-based composites, where inadequate wetting or weak filler–matrix bonding often leads to pull-out, micro-voids and early crack formation, limiting the effective transfer of the inherent stiffness of the carbon phase to the macroscopic composite [27,28].

### 3.2. Electrical Resistivity Measurements

**Figure 4:**
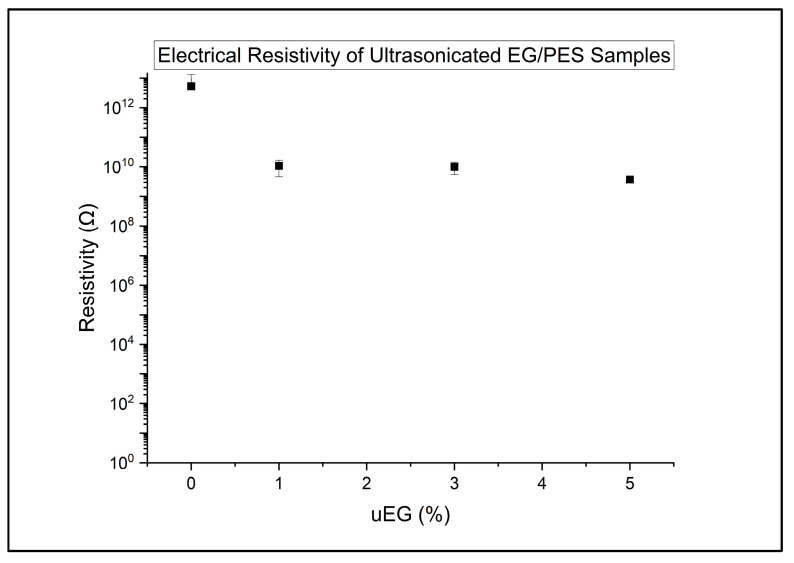
Electrical resistivity of ultrasonicated expanded graphite PES samples.

The surface electrical resistivity of the samples is presented in the following graphs. Results for the uEG samples are presented in Figure 4.

For the uEG-filled composites, the resistivity data show that ultrasonicated expanded graphite on its own is not an especially effective electrical modifier in this system, as shown by the high electrical resistivity in Figure 4. Across 1–5 wt% uEG, the resistivity values remain high (around 10^9^–10^10^ Ω in linear terms), and the uEG group as a whole is not statistically distinguishable from the neat resin or the CF-only group in the ANOVA. Within the uEG family, simple linear regression of the resistivity versus uEG content gives a modest negative slope (about −0.12 decades per 1 wt%), with a reasonably high R^2^ but non-significant *p*-value given the sample size. This indicates a consistent but weak trend towards lower resistivity as more uEG is added, falling far short of a true conductive transition. In percolation terms, the uEG network never approaches a robust, continuous pathway through the thickness; instead, uEG seems to remain mostly as isolated or poorly connected domains that only slightly perturb the insulating matrix.

**Figure 5:**
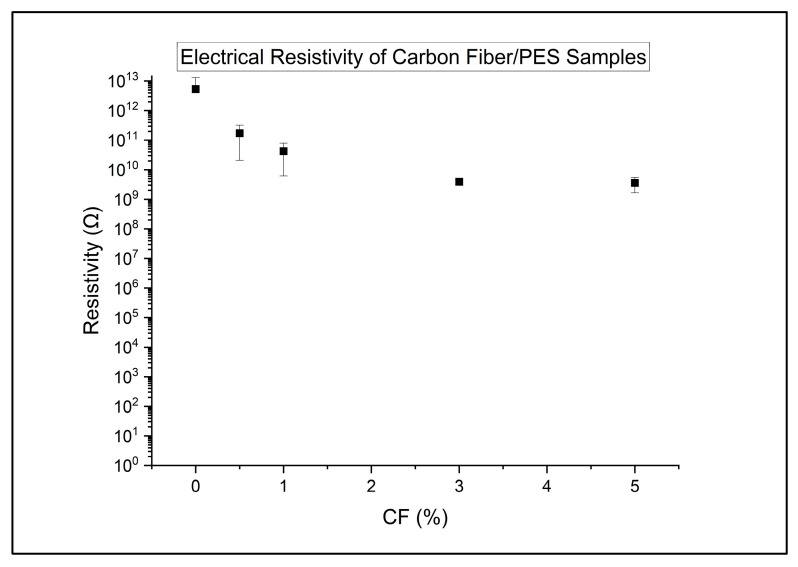
Electrical resistivity of carbon fibre resin samples.

For the CF-containing composites, the effect on electrical resistivity is intermediate, as shown in Figure 5. Moving from 0.5 to 5 wt% CF reduces resistivity from the 10^11^–10^12^ Ω range down into the 10^9^–10^10^ Ω range, but the ANOVA and Tukey comparisons show that, as a group, CF-filled composites are not significantly different from neat or uEG in resistivity. Within the CF family, regression of resistivity versus CF content yields a moderate negative slope of about −0.36 decades per 1 wt% CF (R^2^ ~ 0.82) and a *p*-value close to but above 0.05. This points to a reasonably consistent decrease in resistivity with increasing fibre loading, but the absolute values remain several orders of magnitude higher than those of the vacEG and hybrid systems. In other words, CF alone shifts the material from a highly insulating state to a more weakly insulating or antistatic regime, but does not achieve the kind of percolated, low-resistance network observed when expanded graphite is used under vacuum.

**Figure 6:**
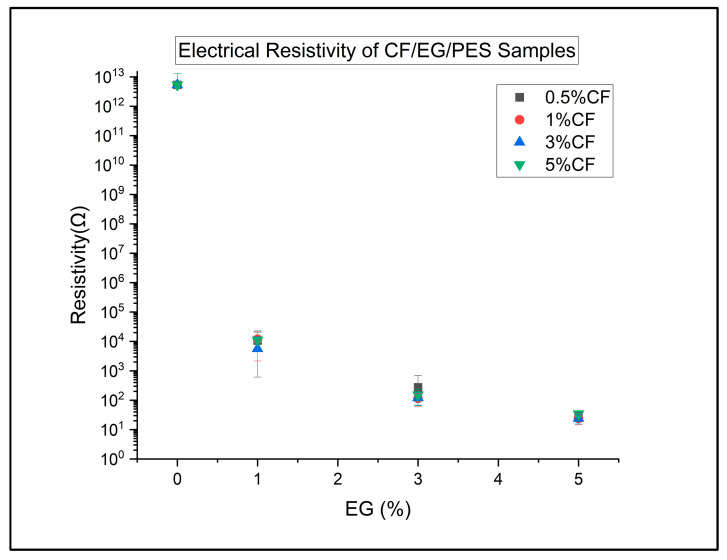
Electrical resistivity of carbon fibre and expanded graphite resin samples. Series are given at constant CF loading.

As shown in Figure 6, the hybrid CF/EG composites occupy the lowest-resistivity region of the dataset and show the clearest signatures of a percolated conductive network, but statistical modelling makes it clear that this behaviour is dominated by the EG content rather than CF. As a group, hybrids and vacEG composites have similar mean resistivities and are not significantly different in the Tukey HSD test, but both groups are vastly more conductive than uEG or CF-filled composites. A multiple linear regression of resistivity versus CF wt% and EG wt% within the hybrid family yields a very strong overall fit (R^2^ ~ 0.94), with a highly significant negative coefficient for EG wt% (about −0.63 decades per 1 wt% EG) and a near-zero, non-significant coefficient for CF wt%. This shows that, once EG is present, varying CF content has almost no systematic effect on resistivity, whereas increasing EG content continues to drive large, predictable reductions in resistivity. Composition by composition, this is reflected in the fact that high-EG hybrids (e.g., 3–5 wt% EG) cluster in the tens-of-Ω range regardless of whether CF is 0.5 or 5 wt%. Overall, the electrical data indicate that EG, particularly when processed under vacuum, is the controlling phase for electrical percolation, while CF’s role in the hybrids is essentially mechanical; it does not significantly alter the conductive network once sufficient EG is present.

**Figure 7:**
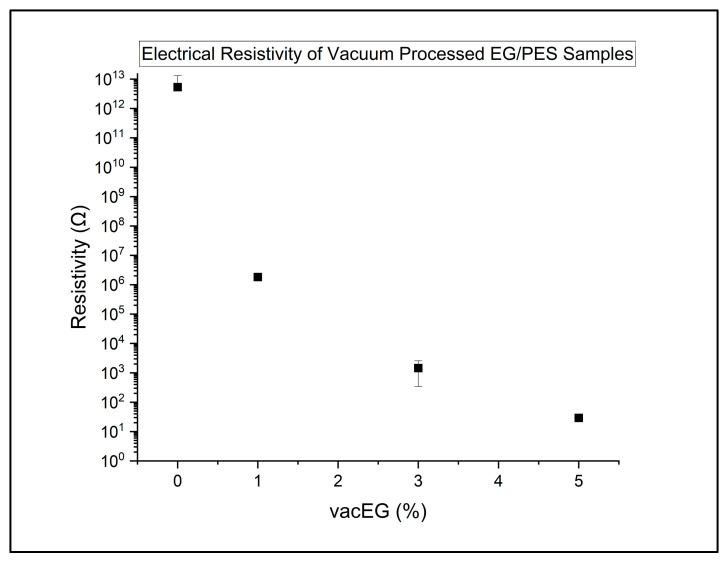
Electrical resistivity of vacuum-processed expanded graphite resin samples.

The vacEG-filled composites behave very differently and are primarily responsible for driving the system into a low-resistivity composite, as illustrated in Figure 7. Compared with the neat resin and uEG family, the vacEG group shows a dramatic downward shift in resistivity by roughly six orders of magnitude, and Tukey’s test confirms that vacEG is significantly more conductive than uEG and CF systems. Within vacEG, regression of resistivity against EG wt% reveals a steep negative slope of about −1.2 decades per 1 wt%, with an excellent fit (R^2^ ~ 0.97). This means that small increments in vacEG content cause very large drops in resistivity, approaching one order of magnitude per wt%. Physically, this suggests that vacuum processing allows the expanded graphite to form a better-connected, more homogeneous conducting network, perhaps by reducing trapped air and improving contact between graphite platelets. By 5 wt% VacEG, the composites have reached resistivities in the tens of Ω, which is characteristic of a well-developed conductive pathway rather than a leaky insulator.

Overall, EG governs conductivity while CF has minimal influence once percolation is established.

### 3.3. Tensile Measurements

**Figure 8:**
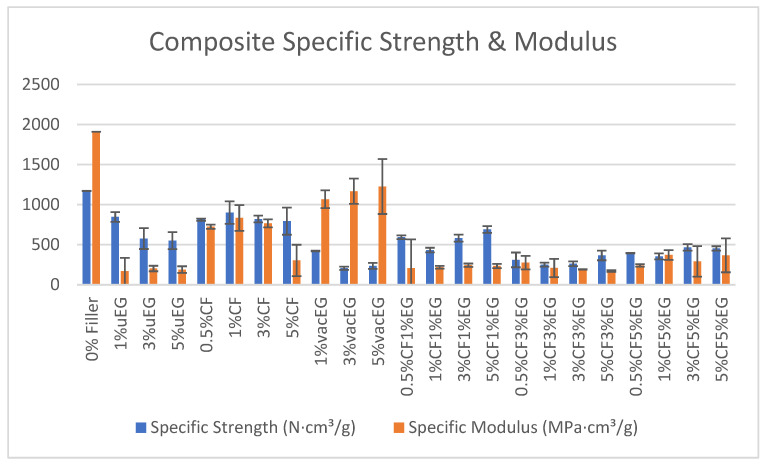
Normalised tensile strength per sample referenced to unfilled PES sample.

Expanded graphite, although excellent for incorporating electrical conductivity into composites, is known to reduce their mechanical strength. Frąc et al. found that the addition of exfoliated expanded graphite caused a decrease in mechanical strength, attributed to possible air incorporation and weak matrix bonding, potentially related to the surfactant used during ultrasonication [13]. Table 1 summarises the tensile measurements for the composites normalised to sample density. In all cases, the use of filler reduced the mechanical strength when compared to the neat resin. Poor interfacial adhesion, filler agglomeration, inadequate dispersion, and increased brittleness can all diminish breaking strength [29,30,31]. To address this issue, the methods in this work employed adequate mixing to minimise agglomeration, vacuum processing to enhance interfacial adhesion, and ultrasonication to decrease particle porosity. Despite these improvements, some causes of the strength decrease were still present. A graph illustrating the specific strength and modulus of the samples is presented in Figure 8.

The mechanical response of the polyester composites is strongly governed by both the type of filler and its loading level. At the group level, analysis of variance was used to compare the five groups of materials: neat polyester, uEG, vacEG, CF and the hybrid CF/EG systems. For each composition, the mean values of max load, tensile modulus, specific strength and specific modulus were used, and a one-way ANOVA was applied to the group means to assess composition-level trends. Where the ANOVA indicated a significant group effect, Tukey’s HSD post hoc test was used to identify which pairs of groups differed significantly.

For the maximum load, the ANOVA showed a highly significant effect of filler type, with neat polyester displaying the highest load-bearing capacity. All three graphite-containing families (uEG, vacEG and hybrids) exhibited substantial reductions in max load relative to the neat matrix, and Tukey’s test confirmed that these reductions were statistically significant. The CF-only composites exhibited high max loads; although the difference relative to the neat resin did not reach statistical significance at α = 0.05 in the Tukey test, the mean max load was numerically lower by ~400 N, indicating a substantial practical reduction. This pattern suggests that, regardless of processing route, expanded graphite tends to introduce defects or stress concentrators that reduce tensile strength, whereas carbon fibre can reinforce the matrix without a significant reduction in maximum load. When EG is combined with CF in the hybrids, the negative influence of EG dominates: hybrid composites show significantly lower max loads than CF-only materials, indicating no positive synergy of combining these two reinforcements from a strength perspective. Comparable observations have been made in CF–graphene hybrids where non-optimised graphene/graphite phases act as additional stress concentrators rather than effective reinforcements [28,32,33].

The tensile modulus exhibits a different, though related, picture. Neat polyester again has the highest modulus in absolute terms, and the ANOVA followed by Tukey HSD confirms that all filled systems are significantly less stiff than the neat polymer. Among the composite groups, however, there is a clear hierarchy: vacEG and CF have significantly higher moduli than both uEG and hybrids, while vacEG and CF do not differ significantly from each other in grouped mean terms. This trend suggests that vacuum processing and carbon fibre addition both provide effective stiffness enhancements relative to uEG and hybrid systems, but neither can restore the modulus to that of the unfilled resin. The fact that uEG and hybrids cluster at low modulus values suggests that dispersion quality, void content, and interfacial adhesion are likely limiting the ability of these systems to form a continuous load-bearing network, whereas the vacEG route and CF alone do so more successfully [28,32,33].

When density is taken into account, the trends in specific strength (strength per unit mass) and specific modulus (stiffness per unit mass) add important nuance. Specific strength is the highest for the neat polyester, followed by CF-only composites, then uEG and hybrids, with vacEG performing the worst. The ANOVA confirms that the neat resin significantly outperforms the graphite-containing samples, CF significantly outperforms vacEG and hybrids, and vacEG samples are significantly weaker than uEG samples on a per-mass basis. This shows that the density reduction associated with vacuum processing is not enough to compensate for the severe loss in tensile strength; in fact, from a structural weight-efficiency standpoint, vacEG is the least favourable option. Hybrids again occupy an intermediate but still degraded regime, reinforcing the view that introducing EG into a CF-reinforced matrix compromises the strength efficiency of the composite; however, this can also be seen as the addition of CF increasing the mechanical performance of EG composites [34].

The specific modulus results are more favourable for graphite, particularly vacEG. Neat polyester retains the highest specific modulus overall and is significantly stiffer per unit mass than all filled systems. However, among the composites, vacEG exhibits by far the highest specific modulus, significantly exceeding CF, uEG and hybrid samples. CF yields a moderate specific modulus, while uEG and hybrid systems have very low values and are not statistically distinguishable from each other. This trend suggests that vacuum-processed samples, despite performing poorly in strength, are relatively successful at delivering mass-efficient stiffness. The combination of moderately high absolute modulus and significantly reduced density appears to make vacEG attractive for stiffness-driven, weight-sensitive applications, albeit at the expense of tensile strength and toughness.

To resolve how these properties evolve with filler percentage, linear regressions were performed within each single-filler family, using filler wt% as the predictor and each mechanical property as the response. In the uEG series (1, 3 and 5 wt% uEG), max load and specific strength both decrease strongly with increasing uEG content, with fitted slopes of roughly −90 N and −74 N·cm/g per 1 wt% uEG, respectively, and relatively high R^2^ values. Although these slopes are not statistically significant at the 0.05 level due to the small sample size (*n* = 3), they indicate a consistent decline in strength and specific strength as more uEG is added. By contrast, the slopes for modulus and specific modulus in the uEG family are close to zero with low R^2^, indicating that within this loading range, uEG content has little systematic effect on stiffness, either in absolute or density-normalised terms. This behaviour is consistent with uEG acting primarily as a defect source rather than an effective stiffening phase.

The vacEG series (1–5 wt%) follows the same qualitative trend for strength: both max load and specific strength decrease with added filler, with negative slopes of approximately −54 N and −47 N·cm/g per 1 wt% EG. The directions and magnitudes are similar to those in the uEG series. For tensile modulus, however, the vacEG slopes are small and slightly negative, suggesting that absolute modulus is relatively high and not strongly dependent on the specific content in this limited range. In sharp contrast, specific modulus in the vacEG family increases almost linearly with EG content, with a large positive slope of around +40 MPa·cm/g per 1 wt% and an R^2^ close to 1. This means that as more VacEG is introduced, the composite becomes markedly stiffer per unit mass, even though its absolute strength and strength efficiency decline. The implication is that the density reduction and geometric arrangement of the expanded graphite under vacuum dominate specific modulus and can be deliberately exploited if stiffness is prioritised over strength.

For the CF-only samples (0.5–5 wt% CF), regression indicates that both max load and specific strength are relatively insensitive to CF content in this range, with small negative slopes and moderate R^2^ values. In other words, once some CF is present, increasing its content from 0.5 to 5 wt% does not yield a strong or consistent gain in load-bearing capacity per specimen or per unit mass in the PES matrix. The behaviour of modulus is more revealing: both absolute and specific moduli show moderately strong negative slopes with CF loading (around −100 MPa and −97 MPa·cm/g per 1 wt% CF, respectively), with reasonably high R^2^. This is largely driven by the pronounced drop in moduli at the highest CF loading (5 wt%), suggesting that over-loading the system with fibres leads to clustering, poor impregnation or void formation, which in turn undermines the stiffness benefit of the reinforcement. Taken together, the single-filler regressions support the idea of optimal, moderate CF content for this matrix, while higher CF or higher EG tends to be detrimental to some combinations of strength and stiffness. The CF-only series shows that modest CF additions can preserve much of the matrix strength, but excessive fibre loadings undermine stiffness, contradicting the classical composite theory. At 1 wt% CF, the specific strength remains high (901 N·cm^3^/g compared to 1171 N·cm^3^/g for the neat resin), and the tensile modulus, though lower than the neat value, is still within a structurally useful range. However, at 5 wt% CF, the tensile modulus drops to 337 MPa, which is substantially below both the neat polymer and the lower-loading CF composites (Table 1). The SEM images of the 5% CF sample (Figure 3a) reveal fibre clusters and pull-out cavities, indicating poor impregnation and interfacial debonding at high local fibre volume fractions. This behaviour is consistent with reports on CF/graphite and CF/graphene hierarchical composites, where non-optimised nanoscale carbon phases or excessive reinforcement content acts as a stress concentrator rather than an effective stiffener, reducing moduli despite the intrinsic stiffness of the fillers. In well-designed CF/graphene hybrids, improvements in interfacial tailoring (e.g., silanisation of graphene or controlled nanofiller loading) can restore or even enhance stiffness, but such strategies were not implemented here [27,28]. Our results therefore indicate that CF does reinforce the polyester matrix at moderate contents, but that over-loading to 5 wt% in this viscous system leads to clustering-induced defects that dominate the macroscopic modulus response. Thus, rather than contradicting the composite theory, our results highlight the critical role of processing-induced defects and interfacial quality in determining whether stiff fillers actually translate into macroscopic stiffness gains.

The hybrid system, where both CF and EG contents vary, was analysed using multiple linear regression to separate their contributions. For maximum load, the model indicates that increasing CF wt% has a positive effect (around +28 N per 1 wt% CF), while increasing EG wt% has a negative effect (around −39 N per 1 wt% EG). Although neither coefficient reaches conventional statistical significance, their magnitudes are physically meaningful and consistent with the raw data: EG-rich hybrids are weak, and additional CF recovers some, but not all, of the lost strength. A similar pattern emerges for specific strength, where CF contributes a modest positive slope and EG a larger negative slope. These findings allow a clear interpretation: within the hybrid design space, CF plays the role of a strength contributor, whereas EG predominantly acts as a strength-diluting phase.

For moduli in hybrids, the regression results are more subdued. Both CF and EG show small positive coefficients for absolute modulus, but with low R^2^ and non-significant *p*-values, indicating that stiffness in the hybrid region is relatively insensitive to modest variations in CF and EG content when examined on an absolute scale. In contrast, for specific modulus, CF has essentially no meaningful effect, while EG shows a statistically significant positive coefficient. This aligns closely with the trends observed with the vacEG samples: increasing EG content in hybrids tends to increase stiffness per unit mass, even as it undermines strength and strength efficiency. In conceptual terms, one can view the hybrids as existing in a two-dimensional trade-off space, where movement along the CF axis primarily influences strength, and movement along the EG axis primarily influences specific modulus, with strong negative coupling between high EG and strength.

### 3.4. Validation of Tensile Results by ANSYS Simulations

Overall, the combined ANOVA and regression analysis paints a coherent picture of the mechanical properties of the various systems. The neat polyester matrix provides the highest strength and stiffness, serving as a benchmark. CF-only composites are the best compromise among the filled systems, preserving load-bearing capacity and offering moderate stiffness, particularly at moderate fibre contents. Expanded graphite, whether ultrasonicated or vacuum-processed, reduces strength and specific strength, and only vacuum processing provides a clear benefit for specific modulus. Hybrid CF–EG systems do not exhibit synergistic reinforcement; instead, they reflect a tuneable compromise in which higher EG content favours stiffness per weight but degrades strength, while higher CF content counteracts the strength loss at the cost of only modest gains in stiffness.

The results of the tensile tests on the samples were validated through numerical modelling. To perform these simulations, a CAD model of the sample was created, and during the simulation, the CAD model of the gauge length was used. Explicit Dynamics in ANSYS was employed for the simulations, with appropriate boundary conditions applied as tested. The simulation results are given in Table 2 with the rendered gauge length indicating the simulated breaking point, indicated as the maximum stress in red. The FEM was intentionally kept at a homogenised, continuum level. For each composite formulation, the tensile modulus and tensile strength used in the model were taken directly from the experimentally measured mean values in Table 1; the material was represented as an isotropic, linear-elastic solid with a maximum principal-stress failure criterion. No micromechanical schemes were applied to predict composite properties from the constituent phases, because our primary aim was to check the internal consistency of the tensile tests for validation against the experimental benchmark rather than to perform first-principles property prediction.

The ANSYS finite element model (FEM) reproduced the experimental tensile-strength trends across all formulations with high fidelity. Experimental tensile strengths range from approximately 4 to 34 MPa, with corresponding FEM predictions spanning 3.6 to 31 MPa; the mean relative error across all 23 samples is 8.3%, the minimum is 0.09%, and the maximum remains 39.8%.

High-error cases exceeding 15% relative difference are 1% uEG (39.8% error; experimental 24.03 MPa vs. ANSYS 14.46 MPa), 3% uEG (17.9%), 5% uEG (15.0%), 5% vacEG (23.8%), and 5% CF 1% EG (21.1%). In each instance, the model underpredicted strength, rendering it conservative. The neat matrix (0% filler), all CF-only samples, and most CF–EG hybrids at lower EG loadings continue to exhibit errors below 7%, often approaching zero. This selective pattern underscores that the model excels for simpler formulations but struggles with uEG/vacEG and higher-loading CF–EG hybrids.

The primary source of these discrepancies lies in the homogenised material representation, which assumes isotropic or orthotropic effective properties and a simple failure criterion. Real uEG/vacEG systems and CF–EG hybrids exhibit pronounced heterogeneity, filler network percolation, enhanced interfacial bonding, and synergistic reinforcement between carbon fibres and expanded graphite mechanisms that elevate experimental strengths beyond rule-of-mixtures predictions. Calibration against the neat matrix or reference CF compositions exacerbates the following: the model interpolates accurately within that micromechanical regime but extrapolates imperfectly to network-forming hybrids, systematically underpredicting their performance. The linear correlation between experimental mean tensile strength and ANSYS predictions remains very strong at 0.962 across all samples.

Low absolute strengths amplify relative errors in cases like 5% vacEG and 3% vacEG (4.70 MPa and 4.13 MPa experimental), where modest absolute differences (~1 MPa) yield 9–24% errors, a common issue in defect-sensitive composites. Idealised FEM boundary conditions, such as perfect alignment, uniform loading, and defect-free geometry, diverge from experimental realities, including misalignments and grip effects, potentially enabling greater load redistribution in tougher formulations.

### 3.5. X-Ray Diffraction (XRD)

**Figure 9:**
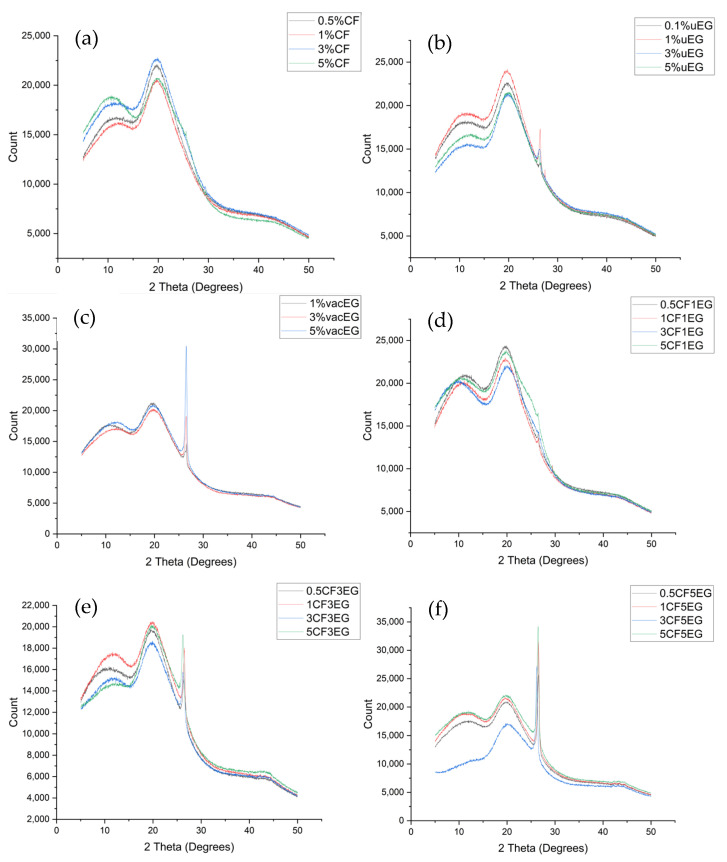
Diffraction pattern of (**a**) carbon fibre-containing samples, (**b**) ultrasonicated expanded graphite samples, (**c**) vacuum-processed expanded graphite samples, (**d**) samples containing both carbon fibre and 1% expanded graphite, (**e**) samples containing carbon fibre and 3% expanded graphite, and (**f**) samples containing carbon fibre and 5% expanded graphite.

XRD was performed on the samples, as illustrated in Figure 9a–f, to investigate filler particle crystallinity differences and changes due to processing.

X-ray diffraction patterns for representative composites are shown in Figure 9a–e. All spectra exhibit a broad amorphous halo centred around 2θ ≈ 18–22°, arising from the unsaturated polyester matrix, and a sharper reflection near 2θ ≈ 26.4–26.6° associated with the (002) planes of graphitic domains. To separate the matrix and filler contributions, a baseline corresponding to the halo was subtracted before calculating the peak parameters of the 002 reflection. Across uEG, vacEG and CF/EG hybrids, the 002 peak position remains close to 26.5° (d_002_ ≈ 0.336 nm), indicating that the average interlayer spacing is broadly consistent with that of expanded graphite and does not undergo large shifts with processing or loading.

The full width at half maximum (FWHM), peak height and integrated area of the 002 reflection were then used as relative indicators of crystalline coherence length, defect density and filler content. For the 1–3 wt% uEG samples, the FWHM values in the range 0.31–0.54° and moderate peak intensities reflect a population of multilayer graphite nanosheets with modest disorder, comparable to those reported in EG-reinforced epoxy nanocomposites processed by sonication and shear mixing. The 5 wt% uEG sample displays a split 002 peak at 26.12° and 26.59°, with FWHM values of 0.46° and 0.38°, respectively, indicating the coexistence of domains with different interlayer spacings and strain states. Similar peak splitting and broadening have been attributed to uneven exfoliation and local restacking of graphite nanosheets in ultrasonicated systems, which generate regions of both highly expanded and more compacted graphite [24,35].

In the vacEG series, the 002 peak remains centred near 26.5° but increases markedly in intensity and area with EG loading, as expected, while FWHM values stay within a relatively narrow window (0.33–0.37°). This suggests that vacuum processing primarily affects the dispersion, packing and connectivity of the EG network at the mesoscale rather than dramatically altering the average crystalline order of the graphitic domains. When EG is combined with CF (CF/EG hybrids), the 002 peak of EG dominates the crystalline response, and contributions from the more turbostratic CF are negligible, in line with other reports on EG- and graphene-modified CF composites, where the graphitic nanofiller gives the main 002 signature [23,28].

Because strain broadening and defect-related effects complicate quantitative Scherrer analysis in heavily processed EG, we use the FWHM mainly as a relative measure of disorder. Nevertheless, applying the Scherrer equation with a shape factor K = 0.9 yields approximate crystallite thicknesses (L_c) of a few tens of nanometres for both uEG and vacEG samples, comparable to values reported for multilayer graphite nanosheets obtained by thermal expansion and ultrasonic exfoliation. The broadening and intensity trends observed here therefore support the microstructural picture drawn from SEM: higher EG loadings and more intensive processing increase the population of disordered, overlapping graphitic domains, which enhances electrical connectivity but also introduces additional potential sites for crack initiation in tension [22,23,24,28,35].

## 4. Comparison with Literature

A number of studies have examined EG or related graphitic fillers in thermoset and thermoplastic matrices for electrical or EMI shielding applications, often reporting low percolation thresholds but providing limited mechanical characterisation. Ramanujam et al. showed that polyphenylene sulphide/expanded graphite (PPS/ExGr) nanocomposites exhibit electrical percolation thresholds as low as 0.5–3 wt% ExGr depending on the processing route, highlighting the strong influence of dispersion quality on network formation. In coal-tar pitch/EG systems, percolation thresholds around 1.5 wt% EG have been reported, again with a focus on conductivity and thermal stability rather than stiffness or strength. Expanded graphite polybenzoxazine nanocomposites have demonstrated conductivities up to ~28 S·cm^−1^ and EMI shielding efficiencies exceeding 50 dB at 15 wt% EG, confirming EG’s potential as an effective shielding filler in high-performance matrices. These results are broadly consistent with our observation that, in a viscous polyester matrix, vacEG and EG-rich hybrids reach surface resistances in the tens of Ω at 3–5 wt% EG, placing them in the conductive regime suitable for EMI shielding and ESD-relevant applications [21,22,23].

From a mechanical perspective, several authors have reported that EG or graphite nanoplatelets can either enhance or degrade stiffness and strength depending on filler loading, matrix viscosity and processing. In anhydride-cured epoxies with 1–2 wt% EG processed by combined sonication and shear mixing, the elastic modulus was increased relative to neat epoxy, attributed to well-dispersed multilayer graphite nanosheets. By contrast, higher graphite loadings or insufficient dispersion have been shown to introduce defects and reduce tensile properties, as observed for some sustainable graphite-reinforced epoxy systems where an optimum around 9 wt% filler maximised tensile and flexural performance before local aggregation became detrimental. Our findings align with this picture: moderate CF or EG contents provide acceptable mechanical performance, but high EG and/or high CF loadings in a viscous polyester lead to clustering, voids and brittle fracture, which suppress both absolute and specific strength [24,27].

Finally, the lack of mechanical synergy observed in our CF/EG hybrids is consistent with several CF/graphene hybrid studies in which nanocarbon phases must be carefully tailored at the fibre–matrix interface to avoid acting as additional stress concentrators. Wang et al. and others have reported large synergetic gains in tensile and flexural strength of CF/epoxy laminates only when graphene or hybrid GnP/CNT coatings were optimised via surface functionalisation and controlled loading; in non-optimised hybrids, graphene agglomerates or poorly bonded platelets reduce interlaminar strength and accelerate delamination. In our hybrids, no specific surface modification or compatibilisation strategy was applied to CF or EG, so the observed behaviour, i.e., EG dominating conductivity, and CF partially recovering strength but not generating true synergy, is in line with the broader literature. Together, these comparisons indicate that the trends identified in this work (low-wt% EG percolation under appropriate processing, strong strength–conductivity trade-offs, and the need for interface engineering to obtain hybrid synergy) are consistent with and extend the existing understanding of EG-based and CF-graphite hierarchical composites [21,22,23,24,27,28,36].

## 5. Conclusions

Neat polyester offers the highest tensile strength and modulus, making it the benchmark for the mechanical properties of composites tested in this study. CF-only composites retain much of their load-bearing capacity and exhibit moderate specific properties; however, stiffness declined at 5 wt% CF due to fibre clustering and inadequate impregnation. Ultrasonicated EG and vacEG both decrease strength and specific strength as EG loading increases, but vacEG achieves a higher specific modulus than uEG because vacuum processing reduces density to ~0.80 g·cm^−3^ while forming a continuous EG skeleton (Figure 3d) that efficiently carries elastic load per unit mass despite crack-initiation sites that limit strength—thanks to its low density and dense EG skeleton.

Electrically, vacEG and CF/EG hybrids show a strong percolation-like transition between 3 and 5 wt% EG, with surface resistances falling by six to ten orders of magnitude and reaching tens of Ω at 5 wt% EG, comparable to conductive EG/polymer systems at similar loadings. In this range, EG mainly governs conductivity, while CF primarily influences mechanical properties. The CF/EG hybrids did not demonstrate true mechanical synergy; instead, they offer a tunable balance where 1–3 wt% CF with 1 wt% EG retains ~70–80% of neat polyester specific strength while achieving substantially lower resistivity, increasing EG content enhances stiffness per unit mass and conductivity but reduces strength, while increasing CF content partially offsets strength loss with only modest effects on stiffness.

For design purposes, our findings suggest that CF-only composites are best suited when tensile strength and structural efficiency are vital and only antistatic or weakly conductive behaviour is required. VacEG and EG-rich hybrids are better suited for applications that prioritise high surface conductivity or EMI shielding over strength, such as conductive inserts, coatings, or secondary structural elements bonded to stiffer substrates. Future work could “pull” this conductivity–strength trade-off using lower-viscosity resins, silane-modified EG/CF surfaces, or small amounts of CNTs to bridge EG platelets, enabling higher conductivity without a drastic loss of strength. These results directly address the knowledge gap identified in the Introduction by mapping mechanical–electrical trade-offs across three processing routes in a single system.

## Figures and Tables

**Table 1:** Tensile measurements of composite samples normalised to sample density.

Sample	Density (g/cm^3^)	Max Load (N)	Elongation at Break (mm)	Tensile Modulus (MPa)	Specific Strength (N·cm^3^/g)	Specific Modulus (MPa·cm^3^/g)
0% Filler	1.16	1354 ± 70.4	3.37 ± 0.41	2210 ± 194	1171.0 ± 60.9	1911.4 ± 167.8
1% uEG	1.13	961 ± 147.5	3.75 ± 0.52	191 ± 39	847.0 ± 130.0	168.3 ± 34.4
3% uEG	1.15	660 ± 120.4	2.88 ± 0.80	232 ± 47	576.4 ± 105.1	202.6 ± 41.0
5% uEG	1.09	599 ± 15.4	3.08 ± 0.03	206 ± 28	551.1 ± 14.2	189.5 ± 25.8
0.5% CF	1.13	918 ± 159.2	3.44 ± 0.41	819 ± 181	812.1 ± 140.8	724.6 ± 160.1
1% CF	1.09	980 ± 46.2	3.57 ± 0.13	907 ± 55	901.3 ± 42.5	834.2 ± 50.6
3% CF	1.12	921 ± 190.2	3.78 ± 0.36	860 ± 220	820.6 ± 169.5	766.3 ± 196.0
5% CF	1.11	882 ± 6.0	1.68 ± 0.08	337 ± 124	793.7 ± 5.4	303.3 ± 111.6
1% vacEG	0.96	405 ± 19.6	1.03 ± 0.03	1026 ± 151	421.5 ± 20.4	1067.9 ± 157.2
3% vacEG	0.8	165 ± 30.1	1.90 ± 0.27	934 ± 274	206.3 ± 37.6	1167.9 ± 342.6
5% vacEG	0.8	188 ± 17.8	1.49 ± 0.07	981 ± 287	235.1 ± 22.3	1226.6 ± 358.9
0.5% CF 1% EG	0.84	498 ± 24.6	2.40 ± 0.04	174 ± 14	593.3 ± 29.3	207.3 ± 16.7
1% CF 1% EG	0.89	386 ± 38.6	2.06 ± 0.13	195 ± 19	432.6 ± 43.3	218.5 ± 21.3
3% CF 1% EG	0.97	563 ± 40.7	2.69 ± 0.11	236 ± 26	581.7 ± 42.0	243.8 ± 26.9
5% CF 1% EG	0.93	644 ± 85.8	2.91 ± 0.39	218 ± 79	691.1 ± 92.1	234.0 ± 84.8
0.5% CF 3% EG	0.73	228 ± 18.4	2.23 ± 0.12	203 ± 84	310.3 ± 25.0	276.3 ± 114.3
1% CF 3% EG	0.68	172 ± 20.4	2.06 ± 0.13	143 ± 2	251.8 ± 29.9	209.3 ± 2.9
3% CF 3% EG	0.74	194 ± 44.7	1.82 ± 0.27	142 ± 8	262.7 ± 60.5	192.3 ± 10.8
5% CF 3% EG	0.73	269 ± 1.1	2.26 ± 0.15	126 ± 11	366.2 ± 1.5	171.5 ± 15.0
0.5% CF 5% EG	0.76	301 ± 28.4	2.22 ± 0.31	184 ± 47	394.7 ± 37.2	241.3 ± 61.6
1% CF 5% EG	0.96	339 ± 39.0	2.20 ± 0.57	356 ± 183	353.6 ± 40.7	371.3 ± 190.9
3% CF 5% EG	0.85	397 ± 22.9	2.15 ± 0.12	249 ± 180	466.5 ± 26.9	292.6 ± 211.5
5% CF 5% EG	0.94	426 ± 75.1	2.60 ± 0.43	345 ± 204	453.8 ± 80.0	367.5 ± 217.3

**Table 2:** Physical and mechanical properties of composites with experimental tensile strength (means) and ANSYS predictions (colour indicating stress, where red indicates the predicted breaking point).

Sample	Tensile Strength (MPa)	Predicted (MPa)	Simulation Render	Relative Error (%)
0% Filler	33.85	31.44	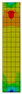	7.11
1% uEG	24.03	14.46	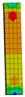	39.82
3% uEG	16.50	13.54	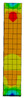	17.92
5% uEG	14.98	12.73	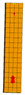	15.02
0.5% CF	22.95	23.45	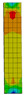	2.16
1% CF	24.50	25.21	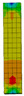	2.80
3% CF	23.03	23.50	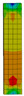	2.03
5% CF	22.05	22.07	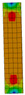	0.09
1% vacEG	10.13	10.97	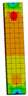	8.29
3% vacEG	4.13	4.51	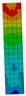	9.20
5% vacEG	4.70	3.58	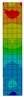	23.80
0.5% CF 1% EG	12.45	12.18	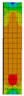	2.17
1% CF 1% EG	9.65	9.48	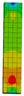	1.70
3% CF 1% EG	14.08	13.57	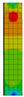	3.63
5% CF 1% EG	16.10	12.70	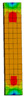	21.11
0.5% CF 3% EG	5.70	5.58	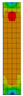	2.10
1% CF 3% EG	4.30	4.27	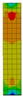	0.60
3% CF 3% EG	4.85	4.98	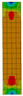	2.68
5% CF 3% EG	6.73	5.81	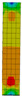	13.67
0.5% CF 5% EG	7.53	7.52	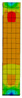	0.13
1% CF 5% EG	8.48	8.80	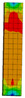	3.70
3% CF 5% EG	9.93	9.26	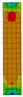	6.70
5% CF 5% EG	10.65	10.97	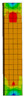	3.40

## Data Availability

The datasets generated during and/or analysed during the current study are available in the following repository reference: https://doi.org/10.5281/zenodo.18625999.

## Abbreviations

The following abbreviations are used in this manuscript:

EG	Expanded Graphite
CF	Carbon Fibre
uEG	Ultrasonicated Expanded Graphite
vacEG/xvacEG	Vacuum-processed Expanded Graphite
PES	Polyester Resin/Polyester (inferred from context)
SEM	Scanning Electron Microscope
XRD	X-Ray Diffraction
ANOVA	Analysis of Variance
HSD	Honest Significant Difference (Tukey’s HSD Test)
FEM	Finite Element Model
FWHM	Full Width at Half Maximum
R^2^	Coefficient of Determination
